# Ruxolitinib mitigates steroid‐refractory CRS during CAR T therapy

**DOI:** 10.1111/jcmm.16176

**Published:** 2020-12-12

**Authors:** Jing Pan, Biping Deng, Zhuojun Ling, Weiliang Song, Jinlong Xu, Jiajia Duan, Zelin Wang, Alex H. Chang, Xiaoming Feng, Yue Tan

**Affiliations:** ^1^ State Key Laboratory of Experimental Hematology National Clinical Research Center for Blood Diseases Institute of Hematology & Blood Diseases Hospital Chinese Academy of Medical Sciences & Peking Union Medical College Tianjin China; ^2^ State Key Laboratory of Experimental Hematology Department of Hematology Beijing Boren Hospital Beijing China; ^3^ Cytology Laboratory Beijing Boren Hospital Beijing China; ^4^ Department of Hematology Beijing Boren Hospital Beijing China; ^5^ Clinical Translational Research Center Shanghai Pulmonary Hospital, Tongji University School of Medicine Shanghai China; ^6^ Central Laboratory Fujian Medical University Union Hospital Fuzhou China

**Keywords:** acute lymphoblastic leukemia, CAR T cell therapy, hematology, immunology, ruxolitinib

## Abstract

Cytokine release syndrome (CRS) and immune effector cell‐associated neurotoxicity are two major CAR T related toxicities. With the interventions of Tocilizumab and steroids, many patients can recover from severe CRS. However, some patients are refractory to steroids and develop life‐threatening consequences. Ruxolitinib is an oral JAKs inhibitor and promising drug in inflammatory diseases. In this pilot study, we evaluate the efficacy of Ruxolitinib in CRS. Of 14 r/r B‐ALL children who received CD19 or CD22 CAR T cell therapies, 4 patients developed severe (≥grade 3) CRS with symptoms that were not alleviated with high‐dose steroids and thus received ruxolitinib. Rapid resolution of CRS symptoms was observed in 4 patients after ruxolitinib treatment. Serum cytokines significantly decreased after ruxolitinib intervention. All patients achieved complete remission on day 30 after infusion, and we could still detect CAR T expansion in vivo despite usage of ruxolitinib. There were no obvious adverse events related to ruxolitinib. In vitro assays revealed that ruxolitinib could dampen CAR T expansion and cytotoxicity, suggesting that the timing and dosage of ruxolitinib should be carefully considered to avoid dampening anti‐leukaemia response. Our results suggest that ruxolitinib is active and well tolerated in steroid‐refractory and even life‐threatening CRS.

## 1 INTRODUCTION

Along with wide application of chimeric antigen receptor (CAR) T therapies in B cell leukaemias and lymphomas, cytokine releasing syndrome (CRS) and immune effector cell‐associated neurotoxicity syndrome (ICANS) are acquainted as two major CAR T cell–related toxicities.^1^ Drugs including Tocilizumab and steroids have been recommended to treat CRS and ICANS. High‐dose steroids are recommended for severe CRS (≥grade 3). High dose of methylprednisolone (1 g/day, equally 15 mg/kg. d) is recommended for refractory CRS.^2^, ^3^ However, life‐threatening consequences may occur if severe CRS cannot be controlled by current regimens. In previous studies, patients with higher leukaemia burden had higher incidence of severe CRS.^4^, ^5^, ^6^, ^7^ In some patients who received CAR T cell therapy after allogenic haematopoietic stem cell transplantation (allo‐HSCT), graft‐versus‐host disease (GVHD) may be complicated with CRS, leading to severe side effect.^8^, ^9^, ^10^

Many key cytokines contribute to inflammation including tumour necrosis factor‐α (TNF‐α), interferon‐γ (INF‐γ), interleukin‐2 (IL‐2) and interleukin‐6 (IL‐6) which bind to receptors that signal via Janus kinases (JAKs; JAK1‐3, Tyk2) and further activate transcription (STAT) family.^11^ Based on their essential roles in transmitting cytokine‐induced signals, the JAKs have become a target for pharmacologic manipulation in many inflammatory diseases.^12^, ^13^, ^14^, ^15^ Ruxolitinib is an oral JAK1/2 inhibitor and a promising drug in inflammatory diseases. Pre‐clinical studies about the therapeutic effects of ruxolitinib on haemaphagocytic lymphohistiocytosis (HLH) have been reported, and related clinical trials are ongoing.^19^, ^20^, ^21^ Ruxolitinib has also been approved for steroid‐refractory (SR) acute GVHD in 2019. Cytokines down‐regulated by ruxolitinib in patients with acute GVHD were mainly IL‐1, IL‐6, TNF‐α and IFN‐γ,^16^, ^17^, ^18^ corresponding to inflammatory effectors that mediated tissue damage and inflammation in CRS and ICANS during CAR T cell therapy. Therefore, in patients who suffer severe CRS during CAR T therapy, ruxolitinib may serve as a promising intervention for controlling life‐threatening consequences, but few studies have reported this thus far.

In this study, we conducted a pilot study to evaluate the effect of ruxolitinib in steroid‐refractory CRS during CAR T therapy. Four patients with steroid‐refractory CRS received ruxolitinib as intervention. Furthermore, in vitro experiments were further conducted for defining the potential mechanisms by which ruxolitinib controls CRS. We aimed to assess the activity and safety of ruxolitinib in children with severe CRS during CAR T cell therapy.

## 2 METHODS

### 2.1 Study design, patients and management of CRS

We performed a pilot study to examine the activity, safety and tolerability of ruxolitinib in treating severe CRS during CAR T cell therapy at Beijing Boren Hospital. The protocol was approved by the institutional review board and was done in accordance with the principles of the Declaration of Helsinki. Patients were enrolled if they were relapsed B acute lymphoblastic leukaemia (B‐ALL) with CD19^+^ or CD22^+^ on blasts. The details of inclusion and exclusion (I/E) criteria are shown in Appendix S1. All enrolled patients received CD19 or CD22 CAR T cell infusion. CD19 and CD22 CAR vectors were previously reported.^5^, ^6^, ^7^, ^22^ Manufacture of CAR T cells, CAR T cell detection and clinical procedures were the same as that previous reported in our previous clinical trials.^5^, ^6^, ^7^ Complete remission or CR with incomplete count recovery (CR/CRi), relapse and minimal residual disease (MRD) were defined in accordance with NCCN clinical practice guidelines in oncology: paediatric acute lymphoblastic leukaemia, version 1.2020.^25^ The sensitivity of the MRD analyses by flow cytometry was 0.01%. Leukaemia‐free survival (LFS) was calculated from date of CAR T cell infusion to date of relapse or death, or the last follow‐up. After CAR T cell infusion, clinical outcomes including overall survival (OS), LFS, adverse effects and relapse were evaluated up to date as of 31 August 2020.

Cytokine releasing syndrome (CRS) and immune effector cell‐associated neurotoxicity syndrome (ICANS) were evaluated and assessed by ASTCT consensus grading system and Management of CAR T cell‐Related Toxicities, NCCN guidelines version 1.2019.^2^, ^3^ Tocilizumab (8 mg/kg/dose) was given when patients had grade 2 CRS, and steroids (equally 3 mg/kg/day methylprednisolone) were given when patients had severe CRS (≥3). The grading of acute GVHD was based on established criteria.^23^ The grading of chronic GVHD was based on the NIH consensus criteria.^24^

### 2.2 Ruxolitinib treatment

Patients received ruxolitinib when they were refractory to high‐dose steroids (equally methylprednisolone 3‐5 mg/kg per day). Ruxolitinib (5 mg twice a day, Novartis, Basel, Switzerland) was orally administrated. Acute haemorrhage was illegible for ruxolitinib treatment. The primary objective was to evaluate the safety and tolerability of ruxolitinib. The secondary objective was to evaluate the efficacy of ruxolitinib in treating severe CRS. Adverse events of ruxolitinib were assessed by CTCAE v5.0.^26^ Platelet transfusion was used for thrombocytopenia and guaranteed the continuous use of ruxolitinib. Corrected count increment (CCI) and percentage platelet response (PPR) were used for evaluating in vivo viability of a transfused platelet.^27^ Successful increment of platelet products were assessed as CCI ≥ 7500 and PPR ≥ 30% at 24 hours. Ruxolitinib was withdrawal when patients had restored from CRS or acute haemorrhage.

### 2.3 Reagents and antibodies in vitro experiments

CD19 CAR T cells were obtained by frozen donor‐derived CD19 CAR T cells. CAR T cells were manufactured with T cells, obtained through RosetteSep™ Human T Cell Enrichment Cocktail (STEMCELL, Vancouver, CA) and transduced with the lentiviral vector expressing pCDH empty vector (#72266, addgene, MA, USA) which were used as negative control. Dexamethasone (DEX) and Ruxolitinib were purchased from MCE (HY‐50856, MCE USA). Nalm 6 cell line (CD19 positive) was purchased from (CRL‐3273, ATCC, Virginia, USA) and cultured in RPMI 1640 (Gibco, NY, USA) supplemented with 10% FCS. CD19 expression on Nalm 6 cells was confirmed by flow cytometry. Monocytes were isolated from donor peripheral blood mononuclear cells (PBMCs) by multi‐analyte flow assay kit (Human CD8/NK Panel, 740267, BioLegend, San Diego, USA). Anti‐human CD3/CD28 monoclonal antibodies (mAb) were purchased from Stemcell (10971, Stemcell, Vancouver, Canada). Flow cytometry was performed using allophycocyanin (APC)‐anti‐human CD19 antibody (392504, BioLegend, San Diego, USA), APC‐anti‐human CD14 antibody (367117, BioLegend, San Diego, USA), APC‐anti‐human CD3 antibody (300311, BioLegend, San Diego, USA), cell surface expression of CD19 CAR was detected by a recombinant human CD19‐Fc chimera protein (789006, BioLegend, San Diego, USA) and a secondary staining of anti‐human IgG Fc‐APC antibody (409603, BioLegend, San Diego, USA ).

### 2.4 In vitro CAR T cell expansion and CRS model

CD19 CAR T cells (1.5 × 10^3^) were stimulated by anti‐CD3/CD28 mAb at for 24 hours. DEX and ruxolitinib were added initially at different concentrations (0, 1 and 10 µmol/L). CD19 CAR T cells were calculated using counting beads (424902, Biolegend, USA) by flow cytometry at 24 hours in different groups. CRS model was established by co‐culturing 5 × 10^4^ Nalm6 cells, 1 × 10^4^ monocytes and 5 × 10^4^ CD19 CAR T cells as published in other group.^28^ The monocytes and CAR T cells were collected from the same donor. DEX and ruxolitinib were added at different concentrations (0, 1 and 10 µmol/L). T cells transduced with pCDH vector were used as a negative control. After 48 hours of coculture, cytokines were monitored in different dosages. The human cytokines included interleukin‐6 (IL‐6), IL‐10, IL‐2, IL‐4, IL‐7a, tumour necrosis factor‐α (TNF‐α), interferon‐γ (INF‐γ), granulysin, granzyme B, granzyme A, Perforin, sFas and sFasL were detected in the culture supernatants after co‐culture using the ‘LEGENDplex Human CD8 Panel’ bead‐based immunoassay (BioLegend, San Diego, USA) according to the manufacturer's protocol. Data were recorded by a FACSCanto II cytofluorometer (Becton Dickinson) and analysed using the ‘LEGENDplex’ Data Analysis software 8.0.

### 2.5 Statistical analysis

Difference of continuous variables between different groups was analysed by unpaired two‐tailed Student's *t* test (cytokines and CRS grade). Difference between chemo‐relapsed and transplant‐relapsed patients was analysed by chi‐square test. The Kaplan‐Meier approach was performed to estimate time‐to‐event analyses. All statistical analyses were performed using NCSS Statistical and Data Analysis version 12.0.2, and all the *P*‐values of < .05 were considered significant.

## 3 RESULTS

### 3.1 Patients enrolled in pilot study

Fourteen patients were enrolled in our study during October 2019 and January 2020, to receive CD19 or CD22 CAR T cell therapy. The flowchart was shown in Figure S1. Eleven patients relapsed after chemotherapy, and 3 patients relapsed after allogenic hematopoietic transplantation (allo‐HCT) of whom one developed moderate chronic GVHD (liver and skin). Six patients were CD19^‐^ relapse after previous CD19 CAR T cell therapy and planned to receive CD22 CAR T cell infusion. The previous times of treatments were 30 (7‐72) months. The detailed characteristics of all patients were in Table 1.

**TABLE 1:** Baseline characteristics of patients

Patient No./Sex/Age	Types of CAR T cells	Ruxo	Fusion gene	Complex Chromosome Aberration	Previous Therapy Period (m)	Baseline Disease Status	Bone marrow blasts by morphology (%)	Bone marrow blasts by FCM (%)^a^	Pre‐HCT	Time to previous transplantation (m)	GVHD before CAR T	Times of tocilizumab using (day)	Max dosage of steroid using (mg/kg/day)^c^	response to CAR T therapy	post CAR treatment	outcome
1/M/16	CD19	Y	E2A‐PBX1	+	16	HR^b^	56	38.06	+	1	‐	0	2.05	CR/MRD‐	‐	CCR/9
2/M/12	CD22	Y	‐	−	38	HR^b^ + EMDs (CNSL)	81.5	65.06	+	3	‐	0	8	CR/MRD‐	‐	CCR/7.5
3/F/9	CD19	Y	E2A‐HLF	−	20	HR^b^	18	11.96	−	#	#	2	18	CR/MRD‐	‐	CCR/9
4/F/18	CD22	Y	‐	+	45	FCM‐MRD + Diffused EMDs	CR	0.04 (78.71% in tissue)	+	25	Chronic	0	5	CR/MRD‐	‐	Death/9
5/M/8	CD19	N	‐	−	39	FCM‐MRD + EMDs (CNSL)	CR	0.2	−	#	#	0	0	CR/MRD‐	HCT	CCR/9
6/M/3	CD19	N	MLL‐AF4	+	13	FCM‐MRD+	CR	1.02	−	#	#	0	0	CR/MRD‐	HCT	CCR/10
7/F/3	CD22	N	‐	+	27	HR^b^	90	80.64	−	#	#	0	1.025	CR/MRD‐	HCT	R/4
8/F/6	CD19	N	‐	−	50	FCM‐MRD+	CR	2.13	−	#	#	1	1.025	CR/MRD‐	HCT	CCR/9
9/M/5	CD19	N	MLL‐AF4	−	30	HR^b^	21.6	6.6	−	#	#	1	1.025	CR/MRD‐	‐	CCR/9
10/M/9	CD19	N	‐	−	54	HR^b^ + EMDs (CNSL)	95	95.46	−	#	#	0	1.025	CR/MRD‐	‐	CCR/8
11/M/9	CD22	N	TEL‐AML1	−	7	HR^b^	32.5	9.75	−	#	#	0	2.05	CR/MRD‐	HCT	CCR/7
12/F/5	CD22	N	‐	+	18	HR^b^	18.2	27.93	−	#	#	0	1.025	CR/MRD‐	HCT	R/2.5
13/M/5	CD22	N	‐	+	42	FCM‐MRD+	CR	6.85	−	#	#	0	0	CR/MRD‐	HCT	CCR/7
14/M/11	CD19	N	‐	−	72	FCM‐MRD+	CR	0.23	−	#	#	0	0	CR/MRD‐	HCT	CCR/11

Abbreviations: #, not applicable or not determined; ‐, no; +, yes; CNSL, central nervous system leukaemia; CR, complete response; EMDs, extramedullary diseases; F, female; FCM, flow cytometry; GVHD, graft‐versus‐host disease; HCT, haematopoietic stem cell transplantation; M, male; MRD, minimal residual disease; Ruxo, ruxolitinib; TL, testicular leukaemia.

^a^ Median marrow blast was determined by marrow biopsy.

^b^ Haematological relapse was defined by ≥ 5% blast determined by marrow biopsy.

^c^ Steroid was calculated by methylprednisolone.

**FIGURE 1:**
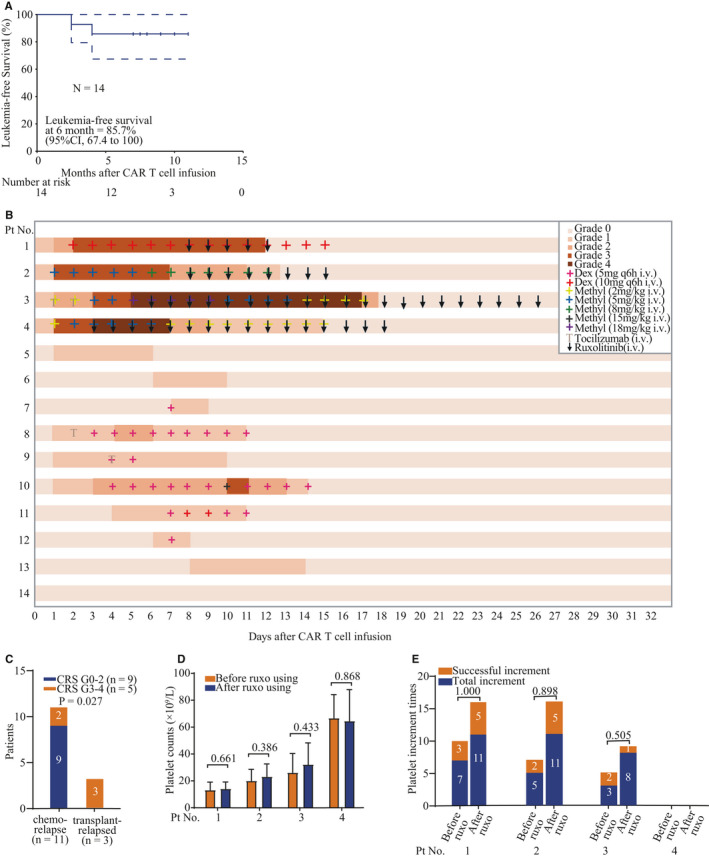
Response to CAR T cell therapy with or without ruxolitinib. A, The response rate in all patients (n = 14), ruxolitinib‐treated patients(n = 4) and non‐ruxolitinib patients (n = 10). Numbers of patients in different groups are indicated in brackets. B, LFS of all patients who received CAR T cell therapy. Dashed lines indicate 95% confidence intervals. Tick marks indicate the time of data censored at the last follow‐up. C, Management of CRS in ruxolitinib‐treated patients. Colours on the swimmer lane plot indicate the highest grade of any cytokine release symptom recorded on each day for patients through the first 32 days after CAR T infusion (n = 4; 0 grade 1‐2 CRS, 4 grade 3‐4 CRS). iv, intravenous. D, Management of CRS in non‐ruxolitinib patients. Colours on the swimmer lane plot indicate the highest grade of any cytokine release symptom recorded on each day for patients through the first 32 days after CAR T infusion (n = 10; 9 grade 1‐2 CRS, 1 grade 3‐4 CRS). iv, intravenous. E, Severity of CRS in no HCT and post‐HCT patients. F, Platelet counts change before and after ruxolitinib treatments in each patient. G, The incidence of successful platelet increments before and after ruxolitinib treatment in each patient. HCT hematopoietic stem cell transplantation. Numbers in parentheses show number of patients with event/number of patients per cohort.

After standard lymphodepleting chemotherapy with fludarabine and cyclophosphamide, autologous CAR T cells were infused in 11 patients without prior HCT, while donor‐derived CAR T cells were infused in 3 post‐HCT patients. The dosages, transduction efficiency and viability of infused CAR T cells were detailed in Table S1. All 14 patients achieved CR/CRi on day 30 after CAR T cell infusion including the 4 patients who received Ruxolitinib treatment. At a median observation time of 9 (2.5‐11) months, eight patients were bridged to allo‐HCT. Two patients relapsed at 2.5 and 4 months. One patient died of infection at 9 months. The 6‐month LFS rate was 85.7 (95%CI, 67.4‐100; Figure 1A).

Five of 14 patients developed severe CRS (≥grade 3), and one patient had recovered after steroid treatment. Four patients were refractory to steroid and therefore received ruxolitinib intervention (Figure S1). One patient had relapsed after chemotherapy, and she had *BTLA* homozygous mutation (c.A590C/p.N197T) and had cytotoxic T lymphocytes (CTL) degranulation defect. One was post‐HCT with complete donor chimera status before CAR T cell infusion.

### 3.2 Management of CRS and usage of ruxolitinib

Nine of 11 patients without prior HCT (81.2%) develop mild to grade 1 to 2 CRS mostly manifested as fever (<40°C) and hypoxia requiring low‐flow nasal cannula (<6 L/minute). The median onset of CRS occurred on day 1 (range 1‐8), and the median time of the resolution of CRS was on day 11 (range 6‐18) after infusion (Figure 1B). Only 2 of these 11 patients (18.2%) developed severe CRS. One of them (pt 10) alleviated within 24 hours after using 18 mg/kg methylprednisolone on day 10 and totally recovered on day 15 (Figure 1B). The other patient (pt 3) developed grade 3 CRS on day 3, and the symptoms did not alleviate by tocilizumab and 5 mg/kg methylprednisolone. She rapidly developed grade 4 CRS and grade 3 ICANS manifested as high fever (>40°C), severe hypotension which needed multiple vasopressors with hypoxia requiring CPAP, generalized seizure and could only be awakens only to tactile stimulus on day 5 (Figure S2). With combined administration of ruxolitinib and high‐dose methylprednisolone on day 6, her fever had remitted within 24 hours and all symptoms started to be alleviated on days 6‐10. Methylprednisolone was gradually reduced and stopped gradually after she had only required low‐flow nasal cannula (<6 L/minute) on days 10‐17 (Figure 1B).

All 3 patients with a prior history of HCT (100%) developed severe CRS (Figure 1B). There was significant difference in median CRS severity between patients without and with a prior HCT (*P* = .027; Figure 1C). All these 3 patients quickly developed grade 3 CRS on days 1‐3 after infusion. One patient with completed donor chimera was also accompanied with active grade III GVHD in skin. All these patients were refractory to steroids and therefore received ruxolitinib. The symptoms in all these 3 patients have been alleviated by combined administration of ruxolitinib and 5 mg/kg methylprednisolone within 24 hours. The median duration of ruxolitinib intervention was 8 days (range 4‐21 days). ICANS was not observed in these 3 patients.

The dose of steroids was reduced after the symptoms were continuously alleviated for 3 days. After withdrawal of steroids, ruxolitinib was reduced to 5 mg per day and withdrawn when patients exhibited no symptoms of CRS. The median duration of ruxolitinib intervention in the 4 patients was 8 days (range 4‐21 days; Figure 1B).

### 3.3 Side effects of ruxolitinib

Platelet reduction has been reported as a major side effect of ruxolitinib.^19^ No patients had active haemorrhage. We monitored the platelet count level in each patient, and there was no significant change after administration of ruxolitinib in the same patient (*P* = .661, .386, .433, .868; Figures 1D and S3A). PPR and CCI were used to assess for ensuring successful increment of platelet products (Figure S3B). The percentage of successful increment of platelet products in each patient was not significantly lower after administration of ruxolitinib (*P* = 1.000, .898, .505; Figure 1E), suggesting that ruxolitinib did not significantly impact platelets. There were no significant manifestations of other side effects that had been reported to be associated with ruxolitinib (Table S2). After withdrawal of ruxolitinib within 30 days, no long‐term side effects were observed.

### 3.4 Cytokines and CAR T cell expansion

**FIGURE 2:**
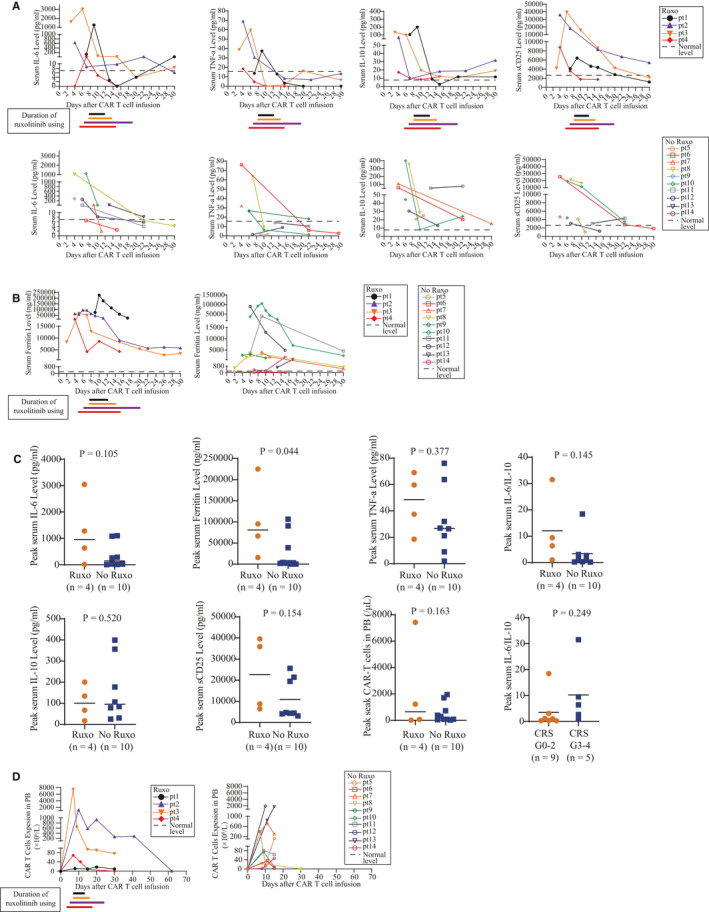
Cytokine profiles and CAR T cell expansion in vivo. A, Serum cytokine profiles (IL‐6, TNF‐α, IL‐10 and sCD25) in PB of 14 patients with and without ruxolitinib treatment after CAR T cell infusion. Different colour represents each individual. Colourful lines indicate duration of ruxolitinib usage in each patient. B, Serum Ferritin levels in PB of 14 patients with and without ruxolitinib treatment after CAR T cell infusion. Different colour represents each individual. Colourful lines indicate duration of ruxolitinib usage in each patient. C, The difference of cytokine profiling levels between ruxolitinib and non‐ruxolitinib subgroup. Horizontal lines indicate medians. D, CAR T cell expansion in PB of 14 patients with and without ruxolitinib treatment after CAR T cell infusion. Colourful lines indicate duration of ruxolitinib usage in each patient

Cytokine profiles were routinely monitored. All patients had elevations of serum cytokine markers of systemic inflammation including interleukin‐6 (IL‐6), interleukin‐10 (IL‐10), soluble CD25 (sCD25) and tumour necrosis factor‐α (TNF‐α) which rapidly reached peak levels at around 5 days after CAR T cell infusion (Figure 2A). Serum ferritin (SF) reflected inflammatory response and high ferritin (>5000 ng/mL) with cytopenia is a criteria for defining severe CRS.^3^, ^29^ All patients treated with ruxolitinib had displayed rapidly rising and high ferritin levels (>15 000 ng/mL) within 10 day after infusion despite the usage of steroids (Figure 2B). The peak levels of ferritin levels were significantly higher in patients with ruxolitinib treatment than those in patients without usage of ruxolitinib (*P* = .042, Figure 2C), but there was no significant difference between the two groups in the peak levels of other cytokines (IL‐6, IL‐10, TNF‐α and sCD25). All cytokine levels decreased rapidly after ruxolitinib intervention.

Ruxolitinib did not completely abrogate CAR T cell expansion in patients, as the CAR T cells are still present in patients after ruxolitinib administration. As the CAR T cells already peaked before ruxolitinib intervention and CAR T cell levels did drop after ruxolitinib intervention, some degree of inhibitory effect of ruxolitinib on CAR T cell expansion could not be excluded. There were no significant differences in peak CAR T levels between two groups of patients (*P* = .594; Figure 2C,D). Due to CAR T cells were not routinely measured, we cannot assess the influence of ruxolitinib on CAR T cell persistence. To the end point, only one patient with ruxolitinib as intervention lost CAR T cell surveillance within 5 months.

### 3.5 Ruxolitinib inhibits CAR T cell expansion and production of some cytokine in vitro

**FIGURE 3:**
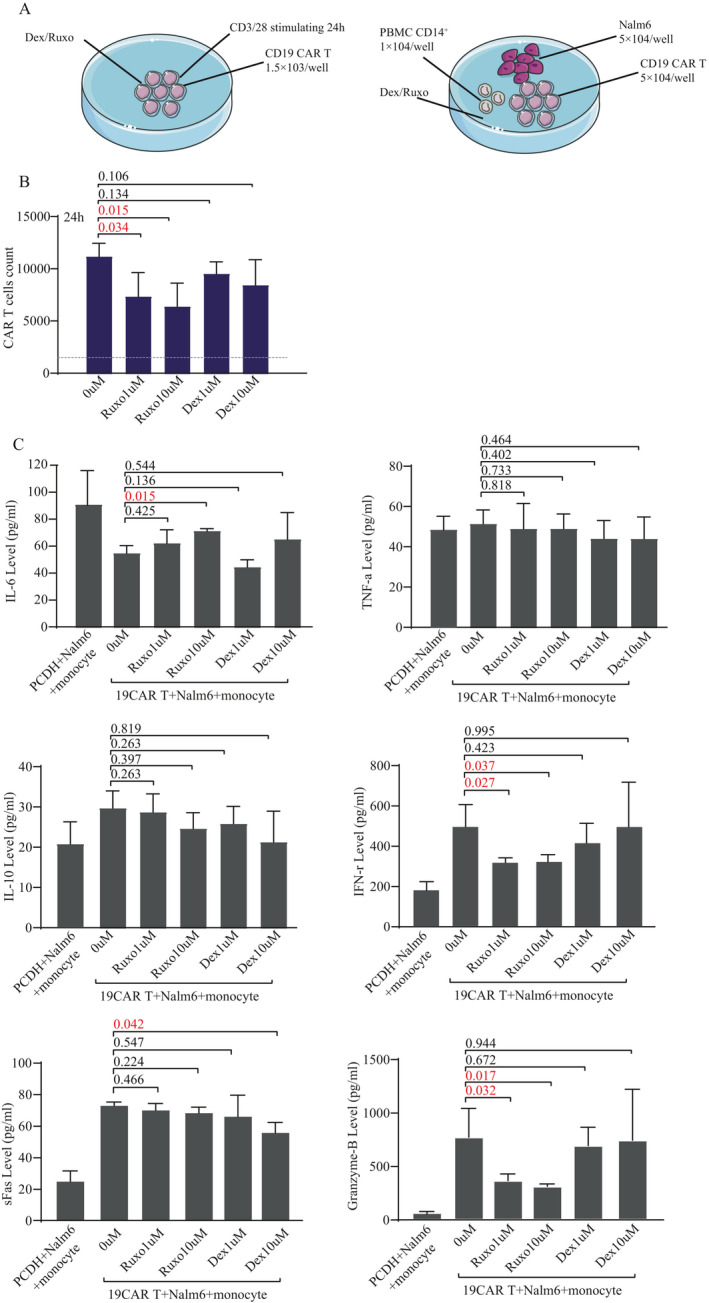
Mechanism of Ruxolitinib and dexamethasone on CAR T cells in vitro. A, CD19 CAR T cell expansion model in vitro and CRS model established by co‐culture with 5 × 10^4^ CD19 CAR T cells, 5 × 10^4^ nalm6 (CD19^+^) and 1 × 10^4^ monocytes in vitro. B, The differences of CAR T cell expansion after 48‐h coculture under different drug concentrations (0, Ruxo 1 umol/L, Ruxo 10 umol/L, Dex 1 umol/L, Dex 10 umol/L). C, The differences of cytokines levels (IL‐6, TNFα, IL‐10, INFγ, sFas and Granzyme B) in supernatant liquid of 48‐hour co‐culture with CD19 CAR T cells, monocytes and Nalm6 cells under different drug concentrations (0, Ruxo 1 umol/L, Ruxo 10 umol/L, Dex 1 umol/L, Dex 10 umol/L), and T cells with pCDH vector, monocytes and Nalm6 cells (CD19^+^) were coculutre as negative control. In B and C panels, the difference is compared by unpaired two‐tailed Student's *t* test. *P*‐values of <.05 were considered significant. Ruxo represents ruxolitinib and Dex represents dexamethasone

We resuscitated frozen donor‐derived CD19 CAR T cells and established CAR T cell expansion and CRS model in vitro (Figure 3A). Firstly, we evaluated the effect of ruxolitinib and DEX on CAR T cell expansion. Compared with the marginal effect of DEX, ruxolitinib could significantly inhibit CD19 CAR T cell expansion at different dosages (*P* = .034 and .015; Figure 3B). Then, we evaluated the impact of ruxolitinib and DEX on cytokine production with an in vitro CRS model by co‐culturing 5 × 10^4^ Nalm6 (CD19^+^) cells, 1 × 10^4^ monocytes and 5 × 10^4^ CD19 CAR T cells.^28^ Different from the impact of DEX, ruxolitinib did not impact the CRS‐related cytokines including IL‐6, IL‐10 and TNF‐a (Figures 3C and S4). However, two cytokines related to T cell cytotoxicity Granzyme‐B and INF‐γ were significantly reduced by different dosages of ruxolitinib.

## 4 DISCUSSION

CRS occurs in 35% to 93% patients after CD19 CAR‐T cell therapy.^30^ The variable incidence and severity of CRS between studies may be due to differences in CAR construct, CAR‐T cell manufacturing, diagnosis, eligibility criteria and the systems used to grade CRS.^30^ Despite the usage of tocilizumab and steroids for the management of severe CRS, there were still 11 of 75 (15%) patients who died from CRS in CD19 CAR T cell study in children and young adults with B‐ALL.^31^ In our previous CD19 and CD22 CAR T cell clinical trials, two of 51 and 4 of 34 patients had died despite using tocilizumab and steroids.^5^, ^6^ Four patients in the current study had developed steroid‐refractory CRS, and our results suggest that ruxolitinib is active and well tolerated in steroid‐refractory CRS. Tocilizumab and steroids were routinely administrated to manage early stage CRS, but the symptoms were not alleviated and even progressed beyond 24 hours in these 4 patients who therefore received ruxolitinib treatment. Rapid resolution of symptoms was observed after ruxolitinib intervention. Severe CRS is a cytokine driven inflammatory syndrome caused by persistent activating CAR T cells. Many T cell activation signalling pathways involve the Janus kinases (JAKs).^20^, ^32^, ^33^ Ruxolitinib which is an oral JAKs inhibitor have been reported to inhibit activation of T lymphocytes and therefore may also be capable of inhibiting activation of CAR T cells. CRS‐related cytokines including IL‐6, IL‐10, sCD25, TNF‐γ and SF quickly decreased in these four patients after ruxolitinib intervention, which implicates that ruxolitinib successfully stops these cytokines release.

Our in vitro experiments indicated that ruxolitinib could not suppress IL‐6 but IL‐6 was rapidly reduced in patients after ruxolitinib administration. This discrepancy may be due to the presence of excretive systems or an intermediate cellular part in human which await future clarification. Our data also showed that ruxolitinib has much stronger impact on CAR T effector function than DEX. Ruxolitinib mainly inhibit CAR T cell expansion and releasing of T cell cytotoxicity related cytokine (INF‐γ and Granzyme B) in vitro. It would be interesting to measure INF‐γ and Granzyme B in patients with CAR T cell therapy in future study. Severe CRS may be controlled by partly via directly inhibiting the hyper‐activation CAR T cells in these four patients. Although they had continued CAR T cell expansion during ruxolitinib intervention, the CAR T cell level quickly dropped after ruxolitinib treatment, implicating that ruxolitinib can suppress CAR T cell expansion in vivo, which was consistent with in vitro analysis. These data also remind us that the usage of ruxolitinib may influence CAR T cell expansion and dampen antitumor effects. Therefore, ruxolitinib should be only considered when patients had life‐threatening CRS and had abundant CAR T cell numbers to guarantee antitumor effects. Moreover, the dose and duration of ruxolitinib administration should be carefully designed.

Major adverse events of ruxolitinib are thrombocytopenia and haemorrhage. Thrombocytopenia is also commonly observed during CAR T therapy. With the help of platelet transfusion, all 4 patients with continuous usage of ruxolitinib did not have active haemorrhage. We compared platelet counts and increment times before and after ruxolitinib to evaluate the influence of ruxolitinib on platelet, and no significantly impact was observed. The frequency of platelet transfusion was also not increased after ruxolitinib intervention. After recovering from CRS, all patients had withdrawn ruxolitinib and platelet counts recovered gradually. No long‐term adverse events were observed. These results suggest that ruxolitinib usage is relatively safe during CAR T therapy with platelet transfusion.

In this study, the 3 patients with prior HCT had developed severe CRS than patients without a prior HCT (*P* = .027). One patient had active chronic GVHD and two patients were very early relapsed (<3 months) with quickly withdrawal immunosuppressive drugs. We think these 3 post‐transplantation patients might have activated donor lymphocytes and other immune cell function before infusion, which may aggravate CRS after infusion of donor‐derived CAR T cells. It indicated that this kind of patients should be carefully managed during CAR T therapy and ruxolitinib is effective in controlling their CRS.

In summary, this study firstly demonstrates a high efficacy and good tolerability of ruxolitinib in managing severe CRS. Ruxolitinib can control steroid‐refractory and life‐threatening CRS. To further evaluate the efficacy and safety of ruxolitinib on severe CRS, our clinical trial is ongoing.

## CONFLICT OF INTEREST

AHC is also a founding member of Shanghai YaKe Biotechnology Ltd. The remaining authors declare no conflict of interest.

## AUTHOR CONTRIBUTION

**Jing Pan:** Methodology (equal); Project administration (equal); Writing‐original draft (equal). **Yue Tan:** Project administration (equal); Visualization (equal); Writing‐review & editing (equal). **Biping Deng:** Resources (supporting). **Zhuojun Ling:** Resources (supporting). **Weiliang Song:** Resources (supporting). **Jinlong Xu:** Resources (supporting). **Jiajia Duan:** Resources (supporting). **Zelin Wang:** Resources (supporting). **Xiaoming Feng:** Funding acquisition (supporting). **Alex H. Chang:** Resources (supporting).

## Supporting information

AppendixS1

## References

[1] Lee DW , Gardner R , Porter DL , et al. Current concepts in the diagnosis and management of cytokine release syndrome. Blood. 2014;124:188‐195. 24876563 10.1182/blood-2014-05-552729 PMC4093680

[2] Daniel WL , Bianca DS , Frederick LL , et al. ASTCT consensus grading for cytokine release syndrome and neurologic toxicity associated with immune effector cells. Biol Blood Marrow Transplant. 2019;25:625‐638. 30592986 10.1016/j.bbmt.2018.12.758 PMC12180426

[3] Thompson JA , Schneider BJ , Brahmer J , et al. Management of immunotherapy‐related toxicities, version 1.2019. J Natl Compr Canc Netw. 2019;17:255‐289. 30865922 10.6004/jnccn.2019.0013

[4] Maude SL , Frey N , Shaw PA , et al. Chimeric antigen receptor T cells for sustained remissions in leukemia. N Engl J Med. 2014;371:1507‐1517. 25317870 10.1056/NEJMoa1407222 PMC4267531

[5] Pan J , Yang JF , Deng BP , et al. High efficacy and safety of low dose CD19 directed CAR‐T cell therapy in 51 refractory or relapsed B acute lymphoblastic leukemia patients. Leukemia. 2017;31:2587‐2593. 28490811 10.1038/leu.2017.145

[6] Pan J , Niu Q , Deng BP , et al. CD22 CAR T cell therapy in refractory or relapsed B acute lymphoblastic leukemia. Leukemia. 2019;33:2854‐2866. 31110217 10.1038/s41375-019-0488-7

[7] Pan J , Zuo SY , Deng BP , et al. Sequential CD19‐22 CAR T therapy induces sustained remission in children With r/r B‐ALL. Blood. 2020;135:387‐391. 31725148 10.1182/blood.2019003293

[8] Hu Y , Wang J , Wei G , et al. A retrospective comparison of allogenic and autologous chimeric antigen receptor T cell therapy targeting CD19 in patients with relapsed/refractory acute lymphoblastic leukemia. Bone Marrow Transplant. 2019;54:1208‐1217. 30518980 10.1038/s41409-018-0403-2

[9] Cruz CR , Micklethwaite KP , Savoldo B , et al. Infusion of donor‐derived CD19‐redirected virus‐specific T cells for B‐cell malignancies relapsed after allogeneic stem cell transplant: a phase 1 study. Blood. 2013;122:2965‐2973. 24030379 10.1182/blood-2013-06-506741 PMC3811171

[10] Brudno JN , Somerville RP , Shi V , et al. Allogeneic T cells that express an anti‐CD19 chimeric antigen receptor induce remissions of B‐Cell malignancies that progress after allogeneic hematopoietic stem‐cell transplantation without causing graft‐versus‐host disease. J Clin Oncol. 2016;34:1112‐1121. 26811520 10.1200/JCO.2015.64.5929 PMC4872017

[11] Kiu H , Nicholson SE . Biology and significance of the JAK/STAT signaling pathways. Growth Factors. 2012;30:88‐106. 22339650 10.3109/08977194.2012.660936 PMC3762697

[12] Kontzias A , Kotlyar A , Laurence A , et al. A new class of kinase inhibitors in cancer and autoimmune disease. Curr Opin Pharmacol. 2012;12:464‐470. 22819198 10.1016/j.coph.2012.06.008 PMC3419278

[13] Laurence A , Pesu M , Silvennoinen O , et al. JAK kinases in health and disease: an update. Open Rheumatol J. 2012;6:232‐244. 23028408 10.2174/1874312901206010232 PMC3460320

[14] Villarino AV , Kanno Y , Ferdinand JR , et al. Mechanisms of JAK/STAT signaling in immunity and disease. J Immunol. 2015;194:21‐27. 25527793 10.4049/jimmunol.1401867 PMC4524500

[15] Cao Y , Wei J , Zou L , et al. Ruxolitinib in Treatment of severe coronavirus disease 2019 (COVID‐19): a multicenter, single‐blind, randomized controlled trial. J Allergy Clin Immunol. 2020;146:137‐146. 32470486 10.1016/j.jaci.2020.05.019 PMC7250105

[16] Madan J , Robert Z , Michael A , et al. Ruxolitinib for the treatment of patients with steroid‐refractory GVHD: an introduction to the REACH trials. Immunotherapy. 2018;10:391‐402. 29316837 10.2217/imt-2017-0156

[17] von Nikolas B , Gabriele I , Olga G , et al. Ruxolitinib in GvHD (RIG) study: a multicenter, randomized phase 2 trial to determine the response rate of ruxolitinib and best available treatment (BAT) versus BAT in steroid‐refractory acute graft‐versus‐host disease (aGvHD) (NCT02396628). BMC Cancer. 2018;18:1132. 30453910 10.1186/s12885-018-5045-7 PMC6245867

[18] Zeiser R , Burchert A , Lengerke C , et al. Ruxolitinib in corticosteroid‐refractory graft‐versus‐host disease after allogeneic stem cell transplantation: a multicenter survey. Leukemia. 2015;29:2062‐2068. 26228813 10.1038/leu.2015.212 PMC4854652

[19] Larisa B , Lauren P , Sridhar R , et al. Ruxolitinib for Treatment of Refractory Hemophagocytic Lymphohistiocytosis. Blood Adv. 2017;1:1533‐1536. 29296794 10.1182/bloodadvances.2017007526 PMC5728466

[20] Albeituni S , Verbist KC , Tedrick PE , et al. Mechanisms of action of ruxolitinib in murine models of hemophagocytic lymphohistiocytosis. Blood. 2019;134:147‐159. 31015190 10.1182/blood.2019000761 PMC6624972

[21] Ahmed A , Merrill SA , Alsawah F , et al. Ruxolitinib in adult patients with secondary haemophagocytic lymphohistiocytosis: an open‐label, single‐centre. Pilot Trial. Lancet Haematol. 2019;6:e630‐e637. 31537486 10.1016/S2352-3026(19)30156-5 PMC8054981

[22] Pan J , Tan Y , Deng BP , et al. Frequent occurrence of CD19‐negative relapse after CD19 CAR T and consolidation therapy in 14 TP53‐mutated r/r B‐ALL children. Leukemia. 2020 10.1038/s41375-020-0831-z. Online ahead of print. 32346068

[23] Przepiorka D , Weisdorf D , Martin P , et al. 1994 consensus conference on acute GVHD grading. Bone Marrow Transplant. 1995;15:825‐828. 7581076

[24] Jagasia MH , Greinix HT , Arora M , et al. National Institutes of Health Consensus Development Project on criteria for clinical trials in chronic graft‐versus‐host disease: I. The 2014 Diagnosis and Staging Working Group report. Biol Blood Marrow Transplant. 2015;21:389‐401. 25529383 10.1016/j.bbmt.2014.12.001 PMC4329079

[25] Brown P , Inaba H , Annesley C , et al.NCCN clinical practice guidelines in oncology: pediatric acute lymphoblastic leukemia, version 1.2020. Accessed 30 May 2019. 10.6004/jnccn.2020.0001 31910389

[26] National Cancer Institute . Common terminology criteria for adverse events (CTCAE). Version 5.0. Available at: https://ctep.cancer.gov/protocolDevelopment/electronic_applications/docs/CTCAE_v5_Quick_Reference_8.5x11.pdf. Accessed July 20, 2018

[27] Nancy WT , Kibet S , Amos M . Determination in vivo viability of a transfused platelet product by corrected count increment and percentage platelet response. Pan Afr Med J. 2017;27:226. 28979628 10.11604/pamj.2017.27.226.12116 PMC5622816

[28] Muneyoshi F , Keisuke S , Satomi K , et al. The novel multi‐cytokine inhibitor TO‐207 specifically inhibits pro‐inflammatory cytokine secretion in monocytes without affecting the killing ability of CAR T cells. PLoS One. 2020;15:e0231896. 32320454 10.1371/journal.pone.0231896 PMC7176125

[29] Döring M , Cabanillas Stanchi KM , Feucht J , et al. Ferritin as an early marker of graft rejection after allogeneic hematopoietic stem cell transplantation in pediatric patients. Ann Hematol. 2016;95:311‐323. 26611853 10.1007/s00277-015-2560-3

[30] Hirayama AV , Turtle CJ . Toxicities of CD19 CAR‐T cell immunotherapy. Am J Hematol. 2019;94:S42‐S49. 30784102 10.1002/ajh.25445

[31] Maude SL , Laetsch TW , Buechner J , et al. Tisagenlecleucel in children and young adults with B‐cell lymphoblastic leukemia. N Engl J Med. 2018;378:439‐448. 29385370 10.1056/NEJMoa1709866 PMC5996391

[32] Rupali D , Peng G , Leslee S , et al. Janus kinase inhibition lessens inflammation and ameliorates disease in murine models of hemophagocytic lymphohistiocytosis. Blood. 2016;127:1666‐1675. 26825707 10.1182/blood-2015-12-684399 PMC4817310

[33] Larisa B , Lauren P , Sridhar R , et al. Ruxolitinib for treatment of refractory hemophagocytic lymphohistiocytosis. Blood Adv. 2017;1:1533‐1536. 29296794 10.1182/bloodadvances.2017007526 PMC5728466

