# Supplementary material for: Ruxolitinib mitigates steroid‐refractory CRS during CAR T therapy
Source: J Cell Mol Med. 2020 Dec 12;25(2):1089–99. doi: 10.1111/jcmm.16176 (PMC7812291; doi:10.1111/jcmm.16176)
Supplement: Supplementary file 1 — AppendixS1 [file JCMM-25-1089-s001.pdf]

Supplemental Materials

Jing Pan, Biping Deng, Zhuojun Ling, Weiliang Song, Jinlong Xu, JiajiaDuan, Zelin Wang, Alex H. Chang, Yue Tan

Table of Contents

**Supplemental methods**	Page
Study design and patients	1
CAR construction, detection and in vitro cytotoxicity	3
Manufacture of CAR T-cells	3
Clinical procedures	3
CAR detection by flow cytometry	3
Management of adverse effect according to symptoms	4
Evaluation time	5
Flow cytometric immunophenotyping	5
**Supplemental tables**	
Supplemental table 1: CAR T-cell treatment information of individual patient	6
Supplemental table 2: Adverse events during ruxolitinib treatment	7
**Supplemental figures**	
Supplemental figure 1	8
Supplemental figure 2	9
Supplemental figure 3	10
Supplemental figure 4	11
**References**	12
**Ethic comment**	13

Supplemental methods

Study design and patients

1.1.1.1 We conducted a pilot study of ruxolitinib on steroid refractory CRS during anti-CD19 (mice derived) or anti-CD22 (humanized) chimeric antigen receptor T cells treating refractory or relapsed B acute lymphoblastic leukemia in Beijing Boren Hospital. The study was approved by the institutional review board of Beijing Boren Hospital, and informed consent was obtained in accordance with the Declaration of Helsinki. All these patients matched the diagnostic criteria for (r/r) B-ALL according to the WHO classification and completed morphological evaluation, immunophenotype analysis by flow cytometry (FCM), cytogenetic analysis by routine G-banding karyotype analysis and leukemia fusion gene screening by multiplex nested reverse transcriptase-polymerase chain reaction (PCR). Patients were eligible if they were heavily treated B-ALL who failed from re-induction chemotherapy after relapse or continued MRD^+ for more than three months, and patients had positive CD19 expression or CD22 expression on leukemia blasts by FCM (>95% CD19 or CD22). Patients are enrolled in ruxolitinib subgroup when they develop severe CRS (>3) or CRS related HLH and symptoms are not controlled by tocilizumab and high dose of steroids (1g/kg per day Methylprednisolone) within 24 hours. Enrolled patients received CD19 or CD22 between October 2019 and January 2020 and were evaluated for responses and adverse effects. After CAR T-cell infusion, clinical outcomes including overall survival (OS), leukemia-free survival (DFS), adverse effects and relapse were evaluated up to date as of August 31th, 2020.

1.2 Inclusion and exclusion criteria for enrolled patients

1.2.1 Inclusion Criteria

1.2.1.1 Patients who were diagnosed as primary refractory or relapsed B-ALL. All the patients matched the diagnostic criteria of ALL according to the WHO classification and conducted morphological evaluation, immunophenotype analysis by flow cytometry (FCM), cytogenetic analysis by routine G-banding karyotype analysis, screen of 56 leukemia-related fusion genes by multiplex nested reverse transcriptase-polymerase chain reaction (RT-PCR), and quantification of fusion genes by real-time PCR with ABL1 as reference. Extramedullary diseases (EMDs) were confirmed $CD19^+$ or $CD22^+$ by FCM and evaluated by positron emission tomography/computed tomography (PET/CT), CT, MRI or ultrasonography. The patient relapsed during chemotherapy or failed from re-induction chemotherapy (including first and second-generation TKIs) after relapse or had a persistent positive minimal residual disease (MRD) for three months. Patients had positive CD19 or CD22 expression on leukemia blasts by FCM (>95% CD19 positive);

1.2.1.2 Age from 0 to 70 years old;

1.2.1.3 Candidates over 18 years old need to be sufficiently conscious and able to sign the treatment consent form and voluntary consent form;

1.2.1.4 Children candidates can be recruited after the legal guardian or patient advocate has signed the treatment consent form and voluntary consent form;

1.2.1.5 Patients are enrolled in ruxolitinib subgroup when they develop severe CRS (>3) or CRS related HLH and symptoms are not controlled by tocilizumab and high dose of steroids (1g/kg per day Methylprednisolone) within 24 hours.

1.1.2 Exclusion criteria

1.1.2.1 Intracranial hypertension or unconscious;

1.1.2.2 Acute heart failure or severe arrhythmia;

1.1.2.3 Acute respiratory failure;

1.1.2.4 Other types of malignant tumors;

1.1.2.5 Diffuse intravascular coagulation;

1.1.2.6 Serum creatinine and/or blood urea nitrogen over 1.5 times than normalrange;

1.1.2.7 Sepsis or other uncontrolled infection;

1.1.2.8 Uncontrolled diabetes mellitus;

1.1.2.9 Severe psychological disorder;

1.1.2.10 Obvious cranial lesions with cranial MRI;

1.1.2.11 More than 20 counts/ul leukemic cells in cerebrospinal fluid;

1.1.2.12 More than 30% leukemic cells in theblood;

1.1.2.13 Stage III WHO/ECOG score;

1.1.2.14 Organ recipients;

1.1.2.15 Pregnant or breastfeeding;

1.1.2.16 Active, uncontrolled infection, including hepatitis B, hepatitis or human immunodeficiency virus (HIV);

1.1.2.17 Ruxolitinib is not given with active hemorrhage;

CAR construction, detection and *in vitro* cytotoxicity

Lentiviral vectors carrying second generation anti-CD19 or anti-CD22 CAR with 4-1BB co-stimulatory and CD3 signaling domains were constructed as previously described.[1,2] Briefly, the CD19 recognition domain was composed of a single-chain fragment variable region derived from the FMC63 monoclonal antibody. The CD22 recognition domain was composed of a single-chain fragment variable region obtained from a human antibody phage display library. Cytotoxicity of CD19 or CD22 CAR T cells have been validated previously[1, 3].

Manufacture of CAR T cells

Peripheral blood (PB) mononuclear cells collected from patients or donor were stimulated with magnetic beads coated withanti-CD3/CD28 antibodies (Life Technologies, Carlsbad, CA, USA; now owned by Thermo Fisher Scientific, Waltham, MA, USA) overnight. The next day, transduction was performed at multiplicity of infection 1:10 ratio. Transduced cells were cultured in X-VIVO 15, a serum-free medium (Lonza) with 300 IU/ml interleukin-2, for the duration of cell culture. Transduction efficiency and cell viability were examined at the time of cell infusion. Transduction efficiency was defined as the ratio of CAR-T to $CD3^+$ T cells, determined by FCM with a proprietary anti-CD19 or anti-CD22 CAR T-cell specific detection reagent. Cell viability was determined by Trypan blue exclusion. When the harvest of CD19 and CD22 CAR T-cells was less than 0.1 10^5/kg, we defined it as CAR manufacture failure. The maximum infused dose of CD19 and CD22 CAR T-cells was 10 10^6/kg.

Clinical procedures

1.2.1.6 CAR T-cells were manufactured from peripheral blood mononuclear cells collected by leukapheresis. Before each CAR T-cell infusion (day 0), patients received lymphodepleting chemotherapy composing of Fludarabine (30 mg/m^2/day) and Cyclophosphamide (250 mg/m^2/day) on days -5 to -3. Patients are enrolled in ruxolitinib subgroup when they develop severe CRS (>3) or CRS related HLH and symptoms are not controlled by tocilizumab and high dose of steroids (1g/kg per day Methylprednisolone) within 24 hours. All patients underwent bone marrow (BM) biopsy examination and radiology studies on days 30 and every month to determine the response and remission status. Bone biopsy, MRD status by FCM and RT-PCR (if the patient had fusion gene), and EMDs evaluation by CT/MRI/PET-CT were also conducted to determine the disease status.

CAR detection by flow cytometry

CD19 or CD22 CAR detection was performed by FCM using biotinylated goat anti-murine or human IgG Fab fragment (Jackson ImmunoResearch Laboratories, West Grove, Pennsylvania) as the first antibody, and Streptavidin conjugated with APC (BD Pharmingen, San Diego, California) for CAR detection. Alternatively, CD19 or CD22 CAR-T cell expansion in vitro and in vivo can be distinguished by flow cytometry assays with proprietary specific CD19 or CD22 CAR-T cell detection reagent (CD19-CAR-Green and CD22-CAR-Green, respectively from Shanghai YaKe Biotechnology Ltd., Shanghai, China). CAR T-cells in cerebrospinal fuild (CSF) were detected when the patients had central nervous system leukemia (CNSL) at enrollment or had signs of neurotoxicity.

Management of adverse effect according to symptoms

Cytokine releasing syndrome (CRS) and neurotoxicity were graded and managed according to ASTCT consensus grading system and the 2019 NCCN guideline about the management of immunotherapy-related toxicities.[3, 4] Patients are enrolled in ruxolitinib subgroup when they develop severe CRS (>3) or CRS related HLH and symptoms are not controlled by tocilizumab and high dose of steroids (1g/kg per day Methylprednisolone) within 24 hours.

Tocilizumab was given according to NCCN guideline, and steroids (2 to 15 mg/kg/d methylprednisolone) were given by intravenous injection (IV) in severe CRS patients (grade≥3). Mannitol (2.5 ml/kg/dose IV), furosemide (1 mg/kg/dose IV) and intrathecal injection of dexamethasone (2-5 mg) were used in patients with neurotoxicity (grade≥2). Other managements had been shown below:

**Symptoms**	**Managements**
Fever	NSAIDs
Myalgia	NSAIDs
VLS; hypotension	Vasopressors
ARDS	CPAP
ARF	Dialysis
AHF	Cardiotonic drugs and diuresis
Dysfunction of liver	Hepatinica and PE (grade 4)
Leukopenia	Protective isolation

Infection	Pathogenic detection and antibiotics use (refer to the Sanford guide to antimicrobial therapy 2016)
Fibrinogenopenia	Replacement of fibrinogen or plasma
HLH/MAS	PE

Abbreviations NSAIDs：non-steroidal anti-inflammatory drugs; VLS: vascular leak syndrome; ARDS: acute respiratory distress syndrome; CPAP: continuous positive airway pressure; ARF: acute renal failure; AHF: acute heart failure; PE: plasma exchange; HLH/MAS: Hemophagocytic Lymphohistiocytosis/Macrophage-activation Syndrome;

Evaluation time

CAR T-cells in the PB were measured on days 0, 1, 7, 15, 30 after each cycle of infusion or as necessary. Serum cytokines were measured on days 0, 1, 7, 15, 30 after each cycle of infusion. To evaluate remission duration, BM biopsy was performed once a month or when the patients had any symptoms of relapse.

Flow cytometric immunophenotyping

The following antibodies were used for flow cytometry based immunophenotype detection: FITC: anti-CD20, anti-CD38, anti-CD15, anti-HLA-DR, anti-CD9, anti-CD7; PE: anti-CD22, anti-CD34, anti-CD10, anti-CD13, anti-CD81, anti-CD123; PerCP: anti-CD45; APC: anti-CD19 (all BD Pharmingen, San Diego, California). For the staining preparation, red blood cells were lysed, and white blood cells were calculated and resuspended in phosphate-buffered saline (PBS) with 2% fetal bovine serum. Samples were analyzed on BD FACS Calibur, collected data were analyzed by FlowJo software (version 10).

Table S1. CAR T-cell treatment information of individual patient

Patient No.	Types of CAR T-cells	Donor/autologous derived	Dosage of CAR T-cells (x10^6/Kg)	CAR T-cell Transduction Efficiency (%)	CAR T-cell Viability (%)
**1**	CD19	Donor	1	33.5	90.4
**2**	CD22	Donor	5.3	67.2	85.2
**3**	CD19	Autologous	5.67	46.2	92.2
**4**	CD22	Donor	6.62	51.1	86.1
**5**	CD19	Autologous	3.76	19.4	83.8
**6**	CD19	Autologous	1.54	22.9	80.6
**7**	CD22	Autologous	3.94	48.2	94.8
**8**	CD19	Autologous	5	56.5	92.1
**9**	CD19	Autologous	5	32.5	87.5
**10**	CD19	Autologous	1	35.3	92.0
**11**	CD19	Autologous	1.95	38.2	83.7
**12**	CD22	Autologous	7.82	34.2	74
**13**	CD22	Autologous	1	60.4	88.6
**14**	CD19	Autologous	5.93	24.6	92

Table S2. Adverse events during ruxolitinib treatment

	Before ruxolitinib			After ruxolitinib		
	**Grade 1-2 （%）**	**Grade 3 （%）**	**Grade 4 （%）**	**Grade 1-2 （%）**	**Grade 3 （%）**	**Grade 4 （%）**
	Number of patients (percent)					
**Blood & Lymphatic System Disorders**						
Anemia	4 (100%)	-	-	4 (100%)	-	-
Febrile neutropenia	4 (100%)	-	-	4 (100%)	-	-
Thrombocytopenia	4 (100%)	-	-	4 (100%)	-	-
Intracranial hemorrhage	-	-	-	-	-	-
Gastrointestinal hemorrhage	-	-	-	-	-	-
**Gastrointestinal Disorders**						
Constipation	-	-	-	-	-	-
Diarrhea	-	-	-	-	-	-
**Infections and Infestations**						
Catheter related infections	-	-	-	-	-	-
Sepsis	-	-	-	-	-	-
**Hepatobiliary disorders**						
Elevated ALT and AST	-	3 (75%)	-	1 (25%)	-	-
Blood bilirubin increased	-	3 (75%)	-	1 (25%)	-	-
**Metabolism and nutrition disorders**						
Hypertriglyceridemia	-	-	-	-	-	-
Hypokalemia	-	-	-	-	-	-
Hyponatremia	-	-	-	-	-	-
Hypophosphatemia	-	-	-	-	-	-
**Respiratory, thoracic and mediastinal disorders**						
Epistaxis	-	-	-	-	-	-
Hypoxia	-	2 (50%)	2 (50%)	-	-	-
Respiratory failure	-	-	-	-	-	-
**Vascular disorders**						
Hypertension	-	-	-	-	-	-
**Neurologic**						
Aphasia	-	-	-	-	-	-
Seizure	-	-	-	-	-	-
Coma	-	-	-	-	-	-

Adverse reaction of ruxolitinib was grading according to CTCAE v5.0. *Abbreviations* ALT: alanine aminotransferase; AST: aspartate aminotransferase.

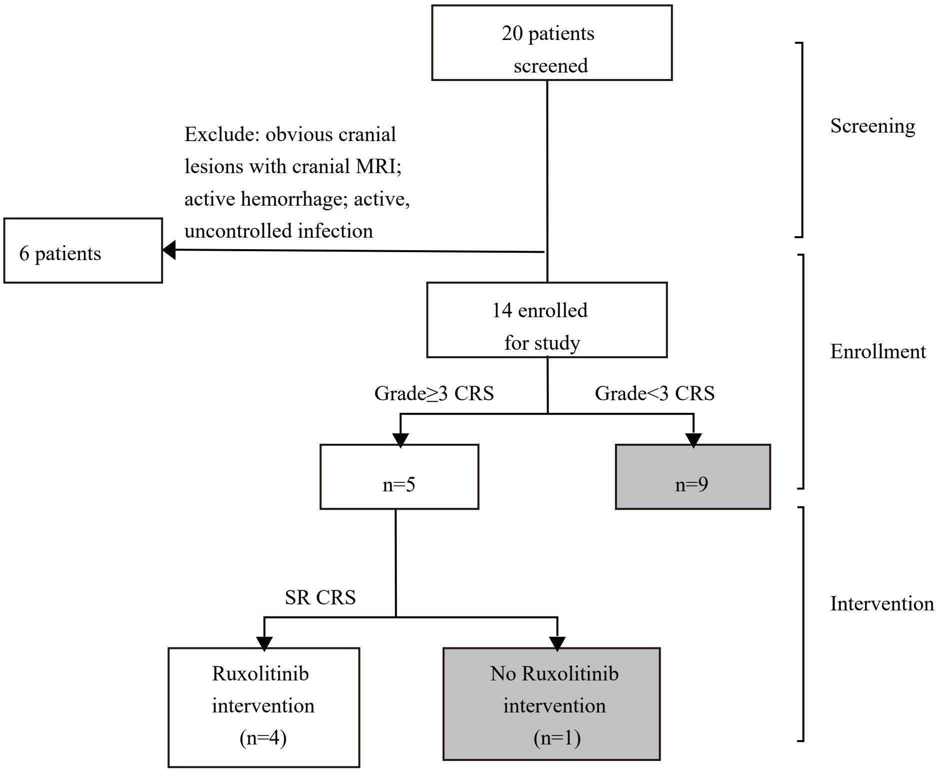

Supplemental Figure 1
Flowchart showing the inclusion of patients in this study.

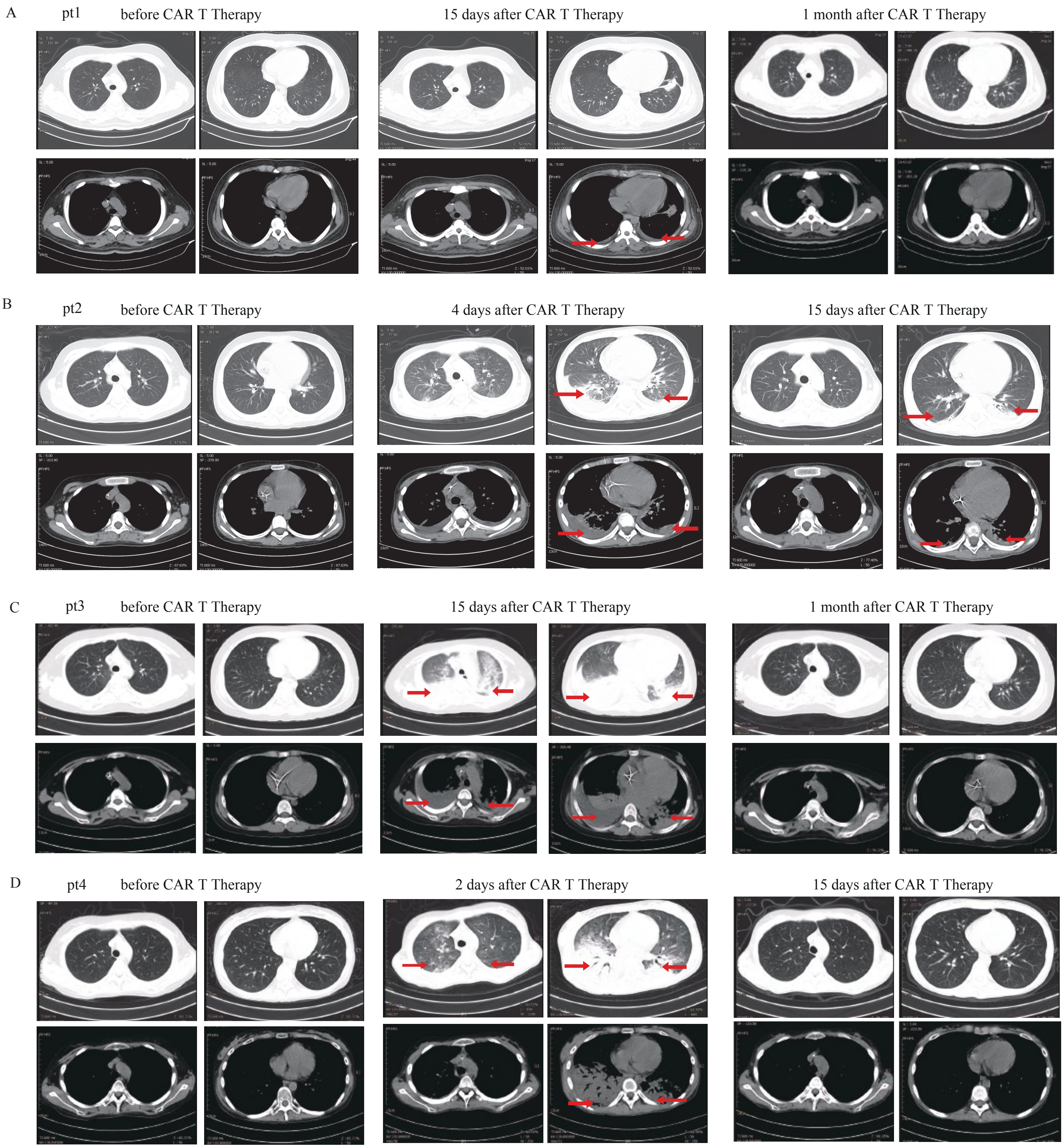

Supplemental Figure 2

Chest CT scan images of all 4 patients in ruxolitinib subgroup before infusion (left panel), on site of CRS (middle panel) and restore from CRS (right panel). Red arrows represent pulmonary edema lesions.

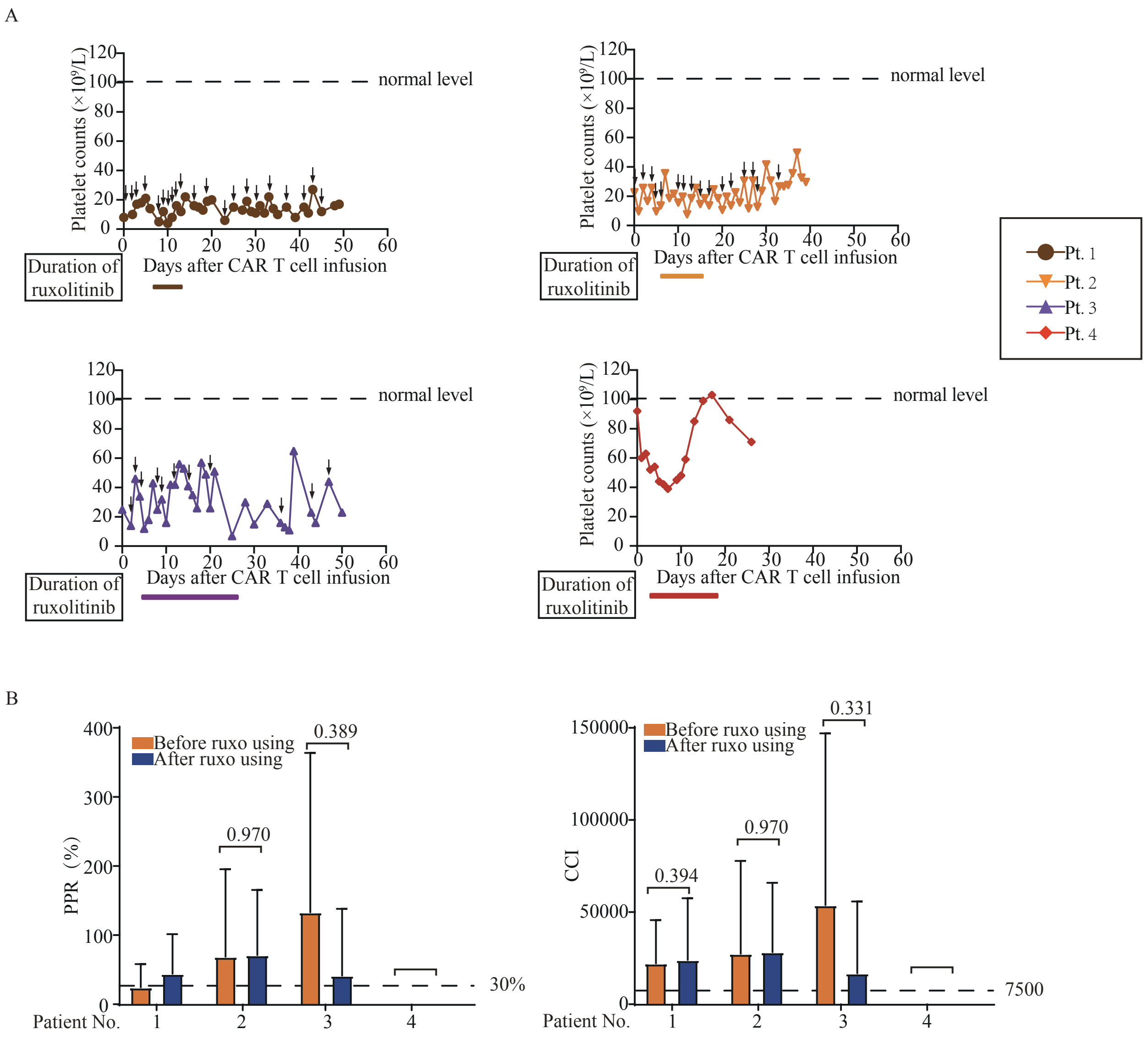

Supplemental Figure 3
(A). Platelet counts of all 4 patients in roxolitinib subgroup after infusion. Black arrows represent platelet transfusion. Normal level is shown as dotted lines. (B). PPR and CCI before and after ruxolitinib treatment in each patient. Normal level is shown as dotted lines. The difference of PPR and CCI for platelet transfusion before and after ruxolitinib treatment is compared by unpaired two-tailed Student's t-test. P-values of <0.05 were considered significant.

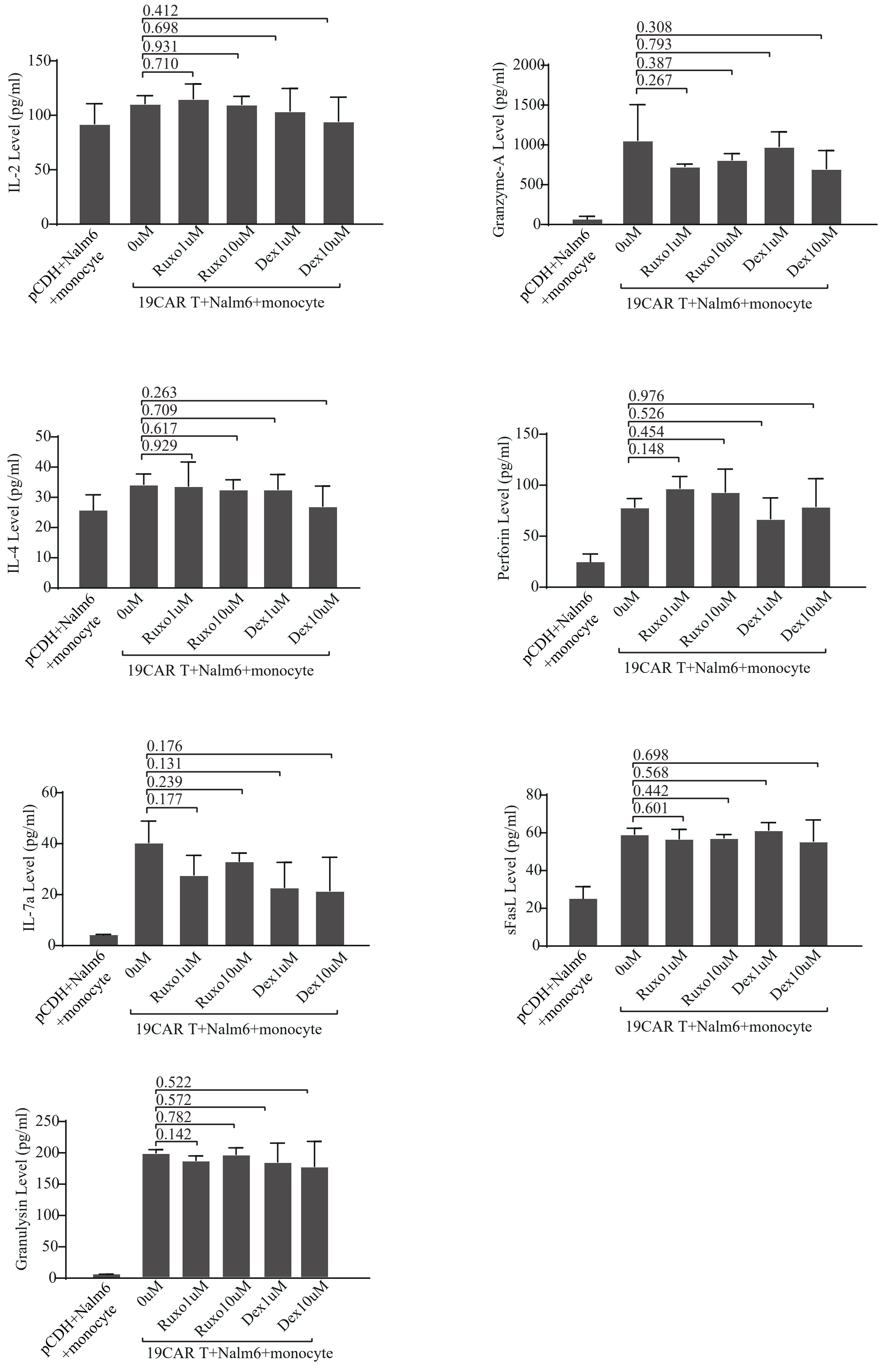

Supplemental Figure 4

The differences of cytokines levels (IL-2, Granzyme A, IL-4, Perforin, IL-7a, sFasL and Granulysin) in supernatant liquid of 48-hour co-culture with CD19 CAR T cells, monocytes and Nalm6 cells ($CD19^+$) under different drug concentrations (0, Ruxo 1uM, Ruxo 10uM, Dex 1uM, Dex 10uM) and T cells with pCDH vector, monocytes and Nalm6 cells ($CD19^+$) were coculutre as negative control. The difference is compared by unpaired two-tailed Student's t-test. P-values of <0.05 were considered significant. Ruxo represents ruxolitinib and Dex represents dexamethasone.

References

1. PanJ, ZuoS, DengB, etal. Sequential CD19-22 CAR T therapy induces sustained remission in children with r/r B-ALL. Blood 10.1182/blood.2019003293. [Epub ahead ofprint]

2. Pan J, Yang JF, Deng BP, et al: High efficacy and safety of low dose CD19 directed CAR-T cell therapy in 51 refractory or relapsed B acute lymphoblastic leukemia patients. Leukemia 31: 2587–93,2017

3. Daniel W Lee, Bianca D Santomasso , Frederick L Locke , Armin Ghobadi, Cameron J Turtle , Jennifer N Brudno, et al. ASTCT Consensus Grading for Cytokine Release Syndrome and Neurologic Toxicity Associated With Immune Effector Cells. Biol Blood Marrow Transplant. 2019 Apr;25(4):625-638.

4. JohnA.Thompson, BryanJ.Schneider, JulieBrahmer, etal: NCCNclinicalpracticeguidelinesinoncology: management of immunotherapy-related toxicities. Version 1, 2019. Accessed date: 11 Mar2019

北京博仁医院医学伦理委员会批文

Beijing Boren Hospital Ethics Committee

北京博仁医院医学伦理委员会批文
Beijing Boren Hospital Ethics Committee

批件编号（Number）	20191025-PJ-001	会议日期（Meeting Date）	2019年10月25日
项目名称（Title）	芦可替尼联合 CAR-T 细胞治疗儿童难治复发急性 B 淋巴细胞白血病预实验<br>A pilot study of combined ruxolitinib and CAR T-cell therapies in r/r B-ALL children		
项目中心（Clinical Center）	北京博仁医院<br>Beijing Boren Hospital		
项目负责人（Principal Investigator）	潘静<br>Jing Pan		
审阅文件 (List of documents were reviewed)	1. 临床研究方案（Clinical Research Program）<br>2. 知情同意书（Informed consent）<br>3. 伦理审查申请表（Application form）		
投票结果:			
同意(Approved)	修正后同意(Conditionally Approved)	不同意(Disapproved)	
5	0	0	
会议结果 (Decision of meeting)	√同意(Approved)<br>修正后同意(Conditionally Approved)<br>不同意(Disapproved)		

评价(Comments):

经伦理委员会审查，同意研究者严格遵守临床研究方案实施，如果意料中发生任何严重不良事件请立即通知本伦理委员会。

伦理委员会主任委员：

北京博仁医院伦理委员会（盖章）

日期：2019 年 10 月 25 日

地址：北京市丰台区郑王坟南 6 号北京博仁医院　邮编：10070
